# Estimation of Large-Dimensional Covariance Matrices via Second-Order Stein-Type Regularization

**DOI:** 10.3390/e25010053

**Published:** 2022-12-27

**Authors:** Bin Zhang, Hengzhen Huang, Jianbin Chen

**Affiliations:** 1 College of Mathematics and Statistics, Guangxi Normal University, Guilin 541004, China; 2 School of Mathematics and Statistics, Beijing Institute of Technology, Beijing 100081, China

**Keywords:** covariance matrix estimation, Stein-type regularization, unbiased estimate

## Abstract

This paper tackles the problem of estimating the covariance matrix in large-dimension and small-sample-size scenarios. Inspired by the well-known linear shrinkage estimation, we propose a novel second-order Stein-type regularization strategy to generate well-conditioned covariance matrix estimators. We model the second-order Stein-type regularization as a quadratic polynomial concerning the sample covariance matrix and a given target matrix, representing the prior information of the actual covariance structure. To obtain available covariance matrix estimators, we choose the spherical and diagonal target matrices and develop unbiased estimates of the theoretical mean squared errors, which measure the distances between the actual covariance matrix and its estimators. We formulate the second-order Stein-type regularization as a convex optimization problem, resulting in the optimal second-order Stein-type estimators. Numerical simulations reveal that the proposed estimators can significantly lower the Frobenius losses compared with the existing Stein-type estimators. Moreover, a real data analysis in portfolio selection verifies the performance of the proposed estimators.

## 1. Introduction

As a fundamental problem in modern multivariate statistics and various practical applications, estimating the covariance matrix of a large-dimensional random vector has attracted significant interest in the last two decades [1,2]. The traditional sample covariance matrix (SCM) becomes unstable and ill-conditioned when the dimension increases proportionally with the sample size. The algorithm, which still employs SCM as the covariance matrix estimator, will result in a drop in performance or failure [3,4]. Although remaining unbiased, the SCM is not a satisfactory estimator of the actual covariance matrix anymore [5,6]. Therefore, it is of great concern to develop well-conditioned covariance matrix estimators in large-dimensional scenarios [7,8,9].

A comprehensive point of view to obtain well-conditioned estimators is by improving the SCM [10]. In the Stein-type regularization (linear shrinkage estimation), a target matrix is preset by rectifying the SCM according to prior information on the covariance structure. For example, the spherical target is a scalar matrix, with the coefficient being the average of the diagonal elements of the SCM [11]. The diagonal target is a diagonal matrix which retainsthe diagonal elements of the SCM [12,13]. Moreover, the Toeplitz-structured target is formed by averaging each diagonal of the SCM [14]. To some extent, the target matrices are covariance matrix estimators. Despite being biased, they usually enjoy low variances. The Stein-type estimator combines the SCM and the target matrix to balance the bias and variance. This method generates a well-conditioned estimator for the spherical target matrix by retaining the sample eigenvectors and shrinking the sample eigenvalues toward their grand mean. Moreover, the Stein-type regularization can be translated as a weighted average between the SCM and the target matrix. Many Stein-type estimators have been developed for various target matrices. Additionally, it is worth mentioning that the optimal Stein-type estimator can be expressed in closed form and significantly outperforms the traditional SCM under some appropriate criteria [15,16,17].

In addition, the nonlinear shrinkage estimation is proposed based on the random matrix theory [18]. By taking spectral decomposition on the SCM, this method retains the sample eigenvectors and estimates the actual eigenvalues by taking a nonlinear transformation on the sample eigenvalues. Then, the nonlinear shrinkage estimator is obtained by assembling the estimated eigenvalues and the sample eigenvectors. In the mean squared error (MSE) sense, the resulting nonlinear shrinkage estimator enjoys a significant advantage over the SCM. It also outperforms the Stein-type estimator for the spherical target. However, both the sample eigenvalues and sample eigenvectors have serious deficiencies in a high-dimensional case [19]. Hence, the existing nonlinear shrinkage strategy, which modifies the sample eigenvalues whileretaining the sample eigenvectors, has some limitations in improving the SCM performance. Moreover, the method can hardly handle the prior structure information employed in the Stein-type regularization. Hence, developing a new nonlinear shrinkage technique is essential to generate outperformed covariance matrix estimators.

This paper combines the SCM and the target matrix via a nonlinear shrinkage strategy to obtain well-conditioned estimators of a large-dimensional covariance matrix. The main contributions are the following:

1. The second-order Stein-type estimator is modeled as a quadratic polynomial concerning the SCM and an almost surely (a.s.) positive definite target matrix. For the spherical and diagonal target matrices, the MSEs between the second-order Stein-type estimator and the actual covariance matrix are unbiasedly estimated under Gaussian distribution.
2. We formulate the second-order Stein-type estimators for the two target matrices as convex quadratic programming problems. Then, the optimal second-order Stein-type estimators are immediately obtained.
3. Some numerical simulations and application examples are provided for comparing the proposed second-order Stein-type estimators with the existing linear and nonlinear shrinkage estimators.

The outline of this paper is as follows. Section 2 proposes the second-order Stein-type estimator based on the Stein-type regularization. In Section 3, the spherical and diagonal matrices are employed as the target matrices. We obtain unbiased estimates of the MSEs between the second-order Stein-type estimators and the actual covariance matrix. The optimal second-order Stein-type estimators are obtained by solving the corresponding optimization problems. Section 4 provides some numerical simulations and two examples to discover the performance of the proposed estimators in large-dimensional scenarios. Section 5 concludes the major work of this paper.

## 2. Notation, Motivation, and Formulation

The symbol $R^{p}$ denotes the set of entire *p*-dimensional real column vectors, $R^{m\times n}$ denotes the set of entire m×n real matrices, and $S^{p}$ denotes the set of entire p×p real symmetric matrices. The bold symbol E denotes the squared matrix having all entries 1 with appropriate dimensions. The symbol $I_{p}$ denotes the p×p identity matrix. For a matrix A, $A^{T}$, tr(A), and ∥A∥, we denote its transpose, trace, and Frobenius matrix norm, respectively. For two matrices A and B, A∘B means their Hadamard (element-wise) product.

Assume that $x_{1},x_{2},\ldots ,x_{n}\in R^{p}$ is an independent and identically distributed (i.i.d.) sample drawn from a certain distribution with mean 0 and covariance matrix Σ. The SCM S is defined by

(1) $$S={\left(s_{ij}\right)}_{p\times p}=\frac{1}{n}\sum_{m=1}^{n}x_{m}x_{m}^{T}.$$

As is widely known, the SCM is ill conditioned in large-dimension scenarios and is even singular when p>n. The Stein-type regularization can produce a well-conditioned covariance matrix estimator based on the SCM [20,21,22,23].

For an a.s. positive definite target matrix T representing the prior information of the covariance structure, the Stein-type estimator combines the SCM and the target matrix with a linear function

(2) f(S,T)=(1−w)S+wT.

Through an equivalent transformation, the expression given by (2) can be recombined as

(3) f(S,T)=S+w(T−S),

where T−S is a regularization term and *w* is a tuning parameter. Moreover, the tuning parameter *w* lies in (0,1] so as to keep the Stein-type estimator a.s. positive definite even when n<p. An interesting fact is that the matrix ${\left(T-S\right)}^{2}$ is still symmetric and positive definite, which motivates us to consider further a quadratic function of the SCM and the target matrix.

For an a.s. positive definite target matrix T, we model the second-order Stein-type estimator of the covariance matrix Σ as

(4) $$\hat{\Sigma }=S+w_{1}\left(T-S\right)+w_{2}{\left(T-S\right)}^{2},$$

where $w_{1},w_{2}$ are the tuning parameters. It is easy to find out that $\hat{\Sigma }\in S^{p}$. In the same manner, we further assume $w_{1}\in \left(0,1\right]$ and $w_{2}\geq 0$ to keep the covariance estimator given by (4) to be a.s. positive definite. We note that the constraint on the tuning parameters is an easy-to-implement condition but is not necessary. One can also consider alternative assumptions, such as the condition number constraint, to obtain positive definite estimators of the large-dimensional covariance matrix [24].

Next, we choose the optimal tuning parameters in (4). In the Stein-type regularization, the MSE between the actual covariance matrix and its estimator is the most commonly used loss function. It includes unknown scalars concerning the expectation operator and the actual covariance matrix. One practical way is to make the MSE available by estimating the unknown scalars and obtaining the optimal shrinkage intensity by minimizing the available MSE [15]. Therefore, we still follow the above steps to find optimal tuning parameters in the second-order Stein-type estimator. To be specific, the loss function of the second-order Stein-type estimator $\hat{\Sigma }$ is defined by

(5) $$M_{T}\left(w|\Sigma \right)=E[∥\hat{\Sigma }-\Sigma {∥}^{2}],$$

where $w={\left(w_{1},w_{2}\right)}^{T}$. Through substituting the expression of $\hat{\Sigma }$ into (5), we can obtain

(6) $$M_{T}\left(w|\Sigma \right)=\frac{1}{2}w^{T}H\left(T\right)w-2w^{T}b\left(T\right)+c,$$

where

(7) $$\begin{array}{cc} H(T) & =2\left[\begin{array}{cc} E[\mathrm{tr}{\left(S-T\right)}^{2}] & -E[\mathrm{tr}{\left(S-T\right)}^{3}]\\ -E[\mathrm{tr}{\left(S-T\right)}^{3}] & E[\mathrm{tr}{\left(S-T\right)}^{4}] \end{array}\right], \end{array}$$

(8) $$\begin{array}{cc} b(T) & =\left[\begin{array}{c} \;E[\mathrm{tr}(S-\Sigma )(S-T)]\\ -E[\mathrm{tr}\left(S-\Sigma \right){\left(S-T\right)}^{2}] \end{array}\right], \end{array}$$

(9) $$\begin{array}{cc} c & =E[\mathrm{tr}{\left(S-\Sigma \right)}^{2}]. \end{array}$$

Therefore, the second-order Stein-type regularization can be modeled as the following optimization problem:

(10) $$\begin{array}{cc} \min & \;\frac{1}{2}w^{T}H\left(T\right)w-2w^{T}b\left(T\right)+c\\ s.t. & \;0\leq e_{1}w\leq 1,\\ \; & \;e_{2}w\geq 0, \end{array}$$

where $e_{1}=\left(1,0\right)$ and $e_{2}=\left(0,1\right)$. It is easy to see that the loss function in problem (10) is a binary quadratic polynomial function about w, and H(T) is the Hessian matrix. By the Cauchy–Schwarz inequality, we have

(11) $${\left(E[\mathrm{tr}{\left(S-T\right)}^{3}]\right)}^{2}\leq E\left[\mathrm{tr}{\left(S-T\right)}^{2}\right]E\left[\mathrm{tr}{\left(S-T\right)}^{4}\right].$$

Therefore, the Hessian matrix H(T) is positive definite. Then, the optimization problem (10) is a convex quadratic program. However, it cannot be solved because the quantities H(T),b(T), and *c* in the objective function are unknown. When the underlying distribution is given and the target matrix is prespecified, we can estimate the unknown quantities H(T),b(T), and *c*. Then, the optimization problem (10) turns out to be available based on plug-in strategy and can be effectively solved.

**Remark** **1.** *The unknown quantity c does not affect the choice of optimal tuning parameter w in the optimization problem (10). Moreover, it is the theoretical MSE between the actual covariance matrix and the classic SCM and plays an important role in evaluating the performance of improved covariance estimators based on the SCM.*

## 3. Optimal Second-Order Stein-Type Estimators

In this section, as the target matrix is specified, we estimate the corresponding unknown quantities under Gaussian distribution, then establish the available version of the optimization problem to obtain the optimal second-order Stein-type estimator.

### 3.1. Target Matrices

As mentioned before, the target matrix represents the prior information of the actual covariance structure. In the Stein-type regularization, the commonly used target matrices include the spherical target, the diagonal target, the Toeplitz-structured target, and the tapered SCM. Among these, the spherical target and the diagonal target are a.s. positive definite, whereas both the Toeplitz-structured target and the tapered SCM are unnecessary. Thereby, in the second-order Stein-type regularization, we employ the spherical target and the diagonal target, given by

(12) $$T_{1}=\frac{\mathrm{tr}(S)}{p}I_{p},\;\;T_{2}=\mathrm{diag}\left(s_{11},\ldots ,s_{pp}\right).$$

The diagonal target $T_{2}$ is also denoted as $D_{S}$ because it consists of the diagonal elements of the SCM.

### 3.2. Available Loss Functions

For the target matrices $T_{1}$ and $T_{2}$, we unbiasedly estimate the loss functions given by (6) through plugging in the estimates of unknown quantities $H\left(T_{i}\right),b\left(T_{i}\right),i=1,2$ and *c* under Gaussian distribution.

First of all, by directly removing the expectation operator, the Hessian matrices $H(T_{i})$ can be estimated by

(13) $$\hat{H}\left(T_{i}\right)=2\left[\begin{array}{cc} \mathrm{tr}{\left(S-T_{i}\right)}^{2} & -\mathrm{tr}{\left(S-T_{i}\right)}^{3}\\ -\mathrm{tr}{\left(S-T_{i}\right)}^{3} & \mathrm{tr}{\left(S-T_{i}\right)}^{4} \end{array}\right].$$

Furthermore, $\hat{H}\left(T_{i}\right),i=1,2$ are, respectively, unbiased estimates of $H(T_{i}),i=1,2$.

Next, for i=1,2, we decompose the unknown vectors $b(T_{i})$ into two terms,

(14) $$\begin{array}{cc} b(T_{i}) & =\left[\begin{array}{c} \;E[\mathrm{tr}\left(S-\Sigma \right)\left(S-T_{i}\right)]\\ -E[\mathrm{tr}\left(S-\Sigma \right){\left(S-T_{i}\right)}^{2}] \end{array}\right]\\ \; & =\left[\begin{array}{c} \;E[\mathrm{tr}\left(S\left(S-T_{i}\right)\right)]\\ -E[\mathrm{tr}\left(S{\left(S-T_{i}\right)}^{2}\right)] \end{array}\right]+\left[\begin{array}{c} -E[\mathrm{tr}\left(\Sigma \left(S-T_{i}\right)\right)]\\ \;\;\;E[\mathrm{tr}\left(\Sigma {\left(S-T_{i}\right)}^{2}\right)] \end{array}\right] \end{array}$$

(15) $$\begin{array}{cc} \; & ≜u\left(T_{i}\right)+v\left(T_{i}\right). \end{array}$$

Similar to the Hessian matrices $H(T_{1})$ and $H(T_{2})$, the first term $u(T_{i})$ can be unbiasedly estimated by

(16) $$\hat{u}\left(T_{i}\right)=\left[\begin{array}{c} \;\mathrm{tr}(S\left(S-T_{i}\right))\\ -\mathrm{tr}(S{\left(S-T_{i}\right)}^{2}) \end{array}\right].$$

Therefore, we only need to estimate the second term $v(T_{i})$. It is challenging to estimate $v(T_{i})$ unbiased because it includes both the expectation operator and the actual covariance matrix Σ. We need the following moment properties about the Wishart distribution [25].

**Lemma** **1.** *Denote A and B as arbitrary symmetric nonrandom matrices, S is the sample covariance matrix given by (1), and then the following equalities hold under Gaussian distribution:*

(17) $$\begin{array}{cc} E[SAS] & =\frac{n+1}{n}\Sigma A\Sigma +\frac{1}{n}\mathrm{tr}\left(\Sigma A\right)\Sigma , \end{array}$$

(18) $$\begin{array}{cc} E[\mathrm{tr}(AS)S] & =\mathrm{tr}\left(A\Sigma \right)\Sigma +\frac{2}{n}\Sigma A\Sigma , \end{array}$$

(19) $$\begin{array}{cc} E[\mathrm{tr}(AS)\mathrm{tr}(BS)] & =\mathrm{tr}\left(A\Sigma \right)\mathrm{tr}\left(B\Sigma \right)+\frac{2}{n}\mathrm{tr}\left(A\Sigma B\Sigma \right). \end{array}$$

By Lemma 1, letting $A=B=I_{p}$, we can obtain

(20) $$E\left[\mathrm{tr}\left(S^{2}\right)\right]=\frac{n+1}{n}\mathrm{tr}\left(\Sigma^{2}\right)+\frac{1}{n}{\mathrm{tr}}^{2}\left(\Sigma \right),\;\;E\left[{\mathrm{tr}}^{2}\left(S\right)\right]={\mathrm{tr}}^{2}\left(\Sigma \right)+\frac{2}{n}\mathrm{tr}\left(\Sigma^{2}\right).$$

Moreover, letting A=Σ and $B=I_{p}$, we have

(21) $$\begin{array}{cc} E[\mathrm{tr}\left(S^{2}\Sigma \right)] & =\frac{n+1}{n}\mathrm{tr}\left(\Sigma^{3}\right)+\frac{1}{n}\mathrm{tr}\left(\Sigma \right)\mathrm{tr}\left(\Sigma^{2}\right), \end{array}$$

(22) $$\begin{array}{cc} E[\mathrm{tr}(S\Sigma )\mathrm{tr}(S)] & =\mathrm{tr}\left(\Sigma \right)\mathrm{tr}\left(\Sigma^{2}\right)+\frac{2}{n}\mathrm{tr}\left(\Sigma^{3}\right). \end{array}$$

Lemma 1 is very helpful to compute the second term $v(T_{i})$ in (14). For the spherical target matrix $T_{1}$, we have

(23) $$E\left[\mathrm{tr}\left(\Sigma \left(S-T_{1}\right)\right)\right]=\mathrm{tr}\left(\Sigma^{2}\right)-\frac{1}{p}{\mathrm{tr}}^{2}\left(\Sigma \right),$$

and

(24) $$\begin{array}{cc} E[\mathrm{tr}\left(\Sigma {\left(S-T_{1}\right)}^{2}\right)]\; & =E\left[\mathrm{tr}\left(S^{2}\Sigma \right)\right]-\frac{2}{p}E\left[\mathrm{tr}\left(S\right)\mathrm{tr}\left(S\Sigma \right)\right]+\frac{1}{p^{2}}E\left[{\mathrm{tr}}^{2}\left(S\right)\right]\mathrm{tr}\left(\Sigma \right)\\ \; & =\frac{np+p-4}{np}\mathrm{tr}\left(\Sigma^{3}\right)+\frac{p^{2}-2np+2}{np^{2}}\mathrm{tr}\left(\Sigma \right)\mathrm{tr}\left(\Sigma^{2}\right)+\frac{1}{p^{2}}{\mathrm{tr}}^{3}\left(\Sigma \right). \end{array}$$

For the diagonal target matrix $T_{2}$, we have

(25) $$E\left[\mathrm{tr}\left(\Sigma \left(S-T_{2}\right)\right)\right]=\mathrm{tr}\left(\Sigma^{2}\right)-\mathrm{tr}\left(D_{\Sigma }^{2}\right),$$

where $D_{\Sigma }=\mathrm{diag}\left(\Sigma_{11},\ldots ,\Sigma_{pp}\right)$. Moreover, we can obtain

(26) $$\begin{array}{cc} \; & E[\mathrm{tr}\left(\Sigma {\left(S-T_{2}\right)}^{2}\right)]\\ \; & =E\left[\mathrm{tr}\left(S^{2}\Sigma \right)\right]-2E\left[\mathrm{tr}\left(ST_{2}\Sigma \right)\right]+E\left[\mathrm{tr}\left(T_{2}^{2}\Sigma \right)\right]\\ \; & =\frac{n+1}{n}\mathrm{tr}\left(\Sigma^{3}\right)+\frac{1}{n}\mathrm{tr}\left(\Sigma \right)\mathrm{tr}\left(\Sigma^{2}\right)-\frac{2n+4}{n}\mathrm{tr}\left(D_{\Sigma }\Sigma^{2}\right)+\frac{n+2}{n}\mathrm{tr}\left(D_{\Sigma }^{3}\right). \end{array}$$

Denote

(27) $$\begin{array}{ccc} a_{1}=\mathrm{tr}\left(\Sigma^{2}\right), & a_{2}={\mathrm{tr}}^{2}\left(\Sigma \right), & a_{3}=\mathrm{tr}\left(D_{\Sigma }^{2}\right),\\ b_{1}=\mathrm{tr}\left(\Sigma^{3}\right), & b_{2}={\mathrm{tr}}^{3}\left(\Sigma \right), & b_{3}=\mathrm{tr}\left(\Sigma \right)\mathrm{tr}\left(\Sigma^{2}\right),\\ c_{1}=\mathrm{tr}\left(D_{\Sigma }^{3}\right), & c_{2}=\mathrm{tr}\left(D_{\Sigma }\Sigma^{2}\right), \end{array}$$

then the vectors $v(T_{i}),i=1,2$ can be rewritten as

(28) $$\begin{array}{cc} v(T_{1}) & =\left[\begin{array}{c} \frac{1}{p}a_{2}-a_{1}\\ \frac{np+p-4}{np}b_{1}+\frac{1}{p^{2}}b_{2}+\frac{p^{2}-2np+2}{np^{2}}b_{3} \end{array}\right], \end{array}$$

(29) $$\begin{array}{cc} v(T_{2}) & =\left[\begin{array}{c} a_{3}-a_{1}\\ \frac{n+1}{n}b_{1}+\frac{1}{n}b_{3}+\frac{n+2}{n}c_{1}-\frac{2n+4}{n}c_{2} \end{array}\right]. \end{array}$$

It is worth noting that each element in $v(T_{i}),i=1,2$ is a linear combination of the quantities in (27). Therefore, we only need to find out the estimates of the quantities in (27).

Firstly, the unbiased estimates of the quantities $a_{i},i=1,2,3$ were proposed in [12,26,27],

(30) $$\begin{array}{cc} \alpha_{1} & =\tau_{a}\left(n\mathrm{tr}\left(S^{2}\right)-{\mathrm{tr}}^{2}\left(S\right)\right),\;\;\alpha_{2}=\tau_{a}\left(\left(n+1\right){\mathrm{tr}}^{2}\left(S\right)-2\mathrm{tr}\left(S^{2}\right)\right),\;\; \end{array}$$

(31) $$\begin{array}{cc} \alpha_{3} & =\tau_{a}\left(n-1\right)\mathrm{tr}\left(D_{S}^{2}\right), \end{array}$$

where $\tau_{a}=\frac{n}{\left(n-1)(n+2\right)}$.

Secondly, denote the matrix W as

(32) $$W=\left[\begin{array}{ccc} n^{2} & 2 & -3n\\ 16 & n^{2}+3n-2 & -6(n+2)\\ -4n & -(n+2) & n^{2}+2n+4 \end{array}\right],$$

Then, the unbiased estimates of $b_{i},i=1,2,3$ can be obtained by the following theorem.

**Theorem** **1.** *Under Gaussian distribution, the following equation holds when n≥3:*

(33) $$E\left[\begin{array}{c} \mathrm{tr}(S^{3})\\ {\mathrm{tr}}^{3}\left(S\right)\\ \mathrm{tr}\left(S\right)\mathrm{tr}\left(S^{2}\right) \end{array}\right]={\left(\tau_{b}W\right)}^{-1}\left[\begin{array}{c} b_{1}\\ b_{2}\\ b_{3} \end{array}\right],$$

*where $\tau_{b}=\frac{n^{2}}{\left(n-1)(n-2)(n+2)(n+4\right)}$.*

**Proof.**  The actual covariance matrix Σ has the spectral decomposition which is described as $\Sigma =\Gamma^{T}\Lambda \Gamma$, where $\Lambda =\mathrm{diag}(\lambda_{1},\ldots ,\lambda_{p})$ is a diagonal matrix consisting of the eigenvalues and Γ is the corresponding unitary matrix. Define $F=\Sigma^{\frac{1}{2}}$, and F is a symmetric matrix; then we have $F^{2}=\Sigma$. For m=1,…,n, denote $z_{m}=F^{-1}x_{m}$, then $z_{m}$ is an i.i.d. sample and $z_{m}∼N\left(0,I_{p}\right)$. Let $X=(x_{1},\ldots ,x_{n})$ and $Z=(z_{1},\ldots ,z_{n})$; then, we have X=FZ. Notice that the SCM is $S=\frac{1}{n}\sum_{m=1}^{n}x_{m}x_{m}^{T}$; therefore, we have

(34) $$nS=XX^{T}=FZZ^{T}F.$$

Moreover, we can obtain

(35) $$\mathrm{tr}\left(nS\right)=\mathrm{tr}\left(FZZ^{T}F\right)=\mathrm{tr}\left(Z^{T}\Sigma Z\right)=\mathrm{tr}\left(Z^{T}\Gamma^{T}\Lambda \Gamma Z\right).$$

Define a matrix $Q^{T}=Z^{T}\Gamma^{T}=\left(q_{1},\ldots ,q_{p}\right)$ and denote $v_{ii}=q_{i}^{T}q_{i}$ and $v_{ij}=q_{i}^{T}q_{j}=q_{j}^{T}q_{i}$ for i,j∈{1,…,p}, then the above equation can be rewritten as

(36) $$\mathrm{tr}\left(nS\right)=\mathrm{tr}\left(Q^{T}\Lambda Q\right)=\sum_{i=1}^{p}\lambda_{i}v_{ii}.$$

In a same manner, we can obtain

(37) $$\begin{array}{c} \mathrm{tr}{\left(nS\right)}^{2}=\sum_{i,j=1}^{p}\lambda_{i}\lambda_{j}v_{ij}^{2},\;{\mathrm{tr}}^{2}\left(nS\right)=\sum_{i,j=1}^{p}\lambda_{i}\lambda_{j}v_{ii}v_{jj}, \end{array}$$

and

(38) $$\begin{array}{cc} \mathrm{tr}{\left(nS\right)}^{3} & =\sum_{i,j,k=1}^{p}\lambda_{i}\lambda_{j}\lambda_{k}v_{ij}v_{ik}v_{jk}, \end{array}$$

(39) $$\begin{array}{cc} {\mathrm{tr}}^{3}\left(nS\right) & =\sum_{i,j,k=1}^{p}\lambda_{i}\lambda_{j}\lambda_{k}v_{ii}v_{jj}v_{kk}, \end{array}$$

(40) $$\begin{array}{cc} \mathrm{tr}\left(nS\right)\mathrm{tr}{\left(nS\right)}^{2} & =\sum_{i,j,k=1}^{p}\lambda_{i}\lambda_{j}\lambda_{k}v_{ij}^{2}v_{kk}. \end{array}$$

By the moment properties of random variables $v_{ii}$ and $v_{ij}$ in [26,28], we have

(41) $$\begin{array}{c} \begin{array}{ccc} E[v_{ii}]=n, & E\left[v_{ii}^{2}\right]=n\left(n+2\right), & E[v_{ij}^{2}]=n,\\ E\left[v_{ii}{ij}^{2}\right]=n\left(n+2\right), & E[v_{ij}v_{ik}v_{jk}]=n & E\left[v_{ii}^{3}\right]=n\left(n+2\right)\left(n+4\right). \end{array} \end{array}$$

where i,j,k are arbitrary mutually unequal numbers in {1, ..., p}. Next, we compute the mathematical expectation of Equations (38)–(40) based on the moment properties in (41). Denote $\mu_{1}=\sum_{i=1}^{p}\lambda_{i}^{3}$, $\mu_{2}=\sum_{i\neq j}^{p}\lambda_{i}^{2}\lambda_{j}$ and $\mu_{3}=\sum_{i\neq j\neq k}^{p}\lambda_{i}\lambda_{j}\lambda_{k}$, where i≠j≠k means that i,j,k are mutually unequal, and we have

(42) $$\begin{array}{cc} E[\mathrm{tr}{\left(nS\right)}^{3}]\; & =n\left(n+2\right)\left(n+4\right)\mu_{1}+3n\left(n+2\right)\mu_{2}+n\mu_{3}, \end{array}$$

(43) $$\begin{array}{cc} E[{\mathrm{tr}}^{3}\left(nS\right)]\; & =n\left(n+2\right)\left(n+4\right)\mu_{1}+3n^{2}\left(n+2\right)\mu_{2}+n^{3}\mu_{3}, \end{array}$$

(44) $$\begin{array}{cc} E[\mathrm{tr}\left(nS\right)\mathrm{tr}{\left(nS\right)}^{2}]\; & =n\left(n+2\right)\left(n+4\right)\mu_{1}+n{\left(n+2\right)}^{2}\mu_{2}+n^{2}\mu_{3}. \end{array}$$

Denote a matrix D as

(45) $$D=\left[\begin{array}{ccc} n(n+2)(n+4) & 3n(n+2) & n\\ n(n+2)(n+4) & 3n^{2}\left(n+2\right) & n^{3}\\ n(n+2)(n+4) & n{\left(n+2\right)}^{2} & n^{2} \end{array}\right],$$

then the above Equations (42)–(44) can be rewritten in the form of a matrix equation:

(46) $$\left[\begin{array}{c} E[\mathrm{tr}{\left(nS\right)}^{3}]\\ E[{\mathrm{tr}}^{3}\left(nS\right)]\\ E[\mathrm{tr}\left(nS\right)\mathrm{tr}{\left(nS\right)}^{2}] \end{array}\right]=D\left[\begin{array}{c} \mu_{1}\\ \mu_{2}\\ \mu_{3} \end{array}\right].$$

Furthermore, the unknown quantities $b_{i},i=1,2,3$ can be decomposed as

(47) $$b_{1}=\mu_{1},\;\;b_{2}=\mu_{1}+3\mu_{2}+\mu_{3},\;\;b_{3}=\mu_{1}+\mu_{2}.$$

Therefore, we have

(48) $$\left[\begin{array}{c} \mu_{1}\\ \mu_{2}\\ \mu_{3} \end{array}\right]={\left[\begin{array}{ccc} 1 & 0 & 0\\ 1 & 3 & 1\\ 1 & 1 & 0 \end{array}\right]}^{-1}\left[\begin{array}{c} b_{1}\\ b_{2}\\ b_{3} \end{array}\right].$$

By the Equations (46) and (48), we have

(49) $$\begin{array}{cc} \left[\begin{array}{c} E[\mathrm{tr}{\left(nS\right)}^{3}]\\ E[{\mathrm{tr}}^{3}\left(nS\right)]\\ E[\mathrm{tr}\left(nS\right)\mathrm{tr}{\left(nS\right)}^{2}] \end{array}\right]\; & =D{\left[\begin{array}{ccc} 1 & 0 & 0\\ 1 & 3 & 1\\ 1 & 1 & 0 \end{array}\right]}^{-1}\left[\begin{array}{c} b_{1}\\ b_{2}\\ b_{3} \end{array}\right]. \end{array}$$

For n≥3, the following equality holds:

(50) $$D{\left[\begin{array}{ccc} 1 & 0 & 0\\ 1 & 3 & 1\\ 1 & 1 & 0 \end{array}\right]}^{-1}={\left(\tau_{b}W\right)}^{-1}.$$

Therefore, we can obtain

(51) $$\begin{array}{c} E\left[\begin{array}{c} \mathrm{tr}{\left(nS\right)}^{3}\\ {\mathrm{tr}}^{3}\left(nS\right)\\ \mathrm{tr}\left(nS\right)\mathrm{tr}{\left(nS\right)}^{2} \end{array}\right]={\left(\tau_{b}W\right)}^{-1}\left[\begin{array}{c} b_{1}\\ b_{2}\\ b_{3} \end{array}\right]. \end{array}$$

This completes the proof. □

Then, the unknown scalars $b_{i},i=1,2,3$ can be unbiasedly estimated by

(52) $$\left[\begin{array}{c} \beta_{1}\\ \beta_{2}\\ \beta_{3} \end{array}\right]=\tau_{b}W\left[\begin{array}{c} \mathrm{tr}(S^{3})\\ {\mathrm{tr}}^{3}\left(S\right)\\ \mathrm{tr}\left(S\right)\mathrm{tr}\left(S^{2}\right) \end{array}\right].$$

**Remark** **2.** *In a large sample scenario, the unknown scalars $b_{i},i=1,2,3$ can be consistently estimated by $\mathrm{tr}(S^{3})$, ${\mathrm{tr}}^{3}\left(S\right)$ and $\mathrm{tr}\left(S\right)\mathrm{tr}\left(S^{2}\right)$ [29]. Theorem 1 shows that these estimates are biased. Moreover, the biases become non-ignorable in high-dimensional situations [30]. Furthermore, by Theorem 1, the biases can be eliminated by the linear combinations of $\mathrm{tr}(S^{3})$, ${\mathrm{tr}}^{3}\left(S\right)$, and $\mathrm{tr}\left(S\right)\mathrm{tr}\left(S^{2}\right)$.*

Thirdly, denote the matrices G, R, and K as follows:

(53) $$\begin{array}{cc} G & =\left(g_{ij}\right)\;\;\mathrm{with}\;\;g_{ij}={∥x^{i}\circ x^{j}∥}^{2}, \end{array}$$

(54) $$\begin{array}{cc} R & =\left(r_{ij}\right)\;\;\mathrm{with}\;\;r_{ij}={∥x^{i}\circ x^{i}\circ x^{j}∥}^{2}, \end{array}$$

(55) $$\begin{array}{cc} K & =\left(k_{ij}\right)\;\;\mathrm{with}\;\;k_{ij}=\left(x^{i}\circ x^{i}\right){\left(x^{i}\circ x^{j}\right)}^{T}, \end{array}$$

where $x^{i}$ is the observations of *i*-th variable for i=1,…,p, then the following theorem holds.

**Theorem** **2.** *Under Gaussian distribution, the following equations hold when n≥3:*

(56) $$\begin{array}{cc} E[n^{3}\mathrm{tr}\left(D_{S}^{3}\right)-3n\mathrm{tr}\left(GD_{S}\right)+2\mathrm{tr}\left(R\right)]\; & =\tau_{c}^{-1}c_{1}, \end{array}$$

(57) $$\begin{array}{cc} E[n^{3}\mathrm{tr}\left(D_{S}S^{2}\right)-2n\mathrm{tr}\left(KS\right)-n\mathrm{tr}\left(GD_{S}E\right)+2\mathrm{tr}\left(RE\right)]\; & =\tau_{c}^{-1}c_{2}, \end{array}$$

*where $\tau_{c}=\frac{1}{n(n-1)(n-2)}$.*

**Proof.**  Let $F=(f_{ij})$, and F is a symmetric matrix; then, we have $F^{2}=\Sigma =\left(\sigma_{ij}\right)$. Therefore, for arbitrary i,j∈{1,…,p}, the equalities $\sigma_{ij}=\sum_{k=1}^{p}f_{ik}f_{jk}$ and $\sigma_{ii}=\sum_{k=1}^{p}f_{ik}^{2}$ hold. For m=1,…,n, denote $x_{m}={\left(x_{m1},x_{m2},\ldots ,x_{mp}\right)}^{T}$ and $z_{m}=F^{-1}x_{m}$, then $z_{m}$ is an i.i.d. sample and $z_{m}∼N\left(0,I_{p}\right)$. Let $z_{m}={\left(z_{m1},z_{m2},\ldots ,z_{mp}\right)}^{T}$, then $z_{mk},m=1,\ldots ,n,k=1,\ldots ,p$ are mutually independent standard Gaussian random variables. For arbitrary m∈{1,…,n} and i,j∈{1,…,p}, we have $x_{mi}=\sum_{k=1}^{p}f_{ik}z_{mk}$ and $x_{mj}=\sum_{k=1}^{p}f_{jk}z_{mk}$. Denote that SCM $S=(s_{ij})$, then $s_{ij}$ can be decomposed as follows:

(58) $$s_{ij}=\frac{1}{n}\sum_{m=1}^{n}x_{mi}x_{mj}=\frac{1}{n}\sum_{m=1}^{n}\sum_{k_{1},k_{2}=1}^{p}f_{ik_{1}}f_{jk_{2}}z_{mk_{1}}z_{mk_{2}}.$$

Then, for arbitrary m∈{1,…,n} and i,j∈{1,…,p}, we have

(59) $$E\left[x_{mi}x_{mj}\right]=\sum_{k_{1},k_{2}=1}^{p}f_{ik_{1}}f_{jk_{2}}E\left[z_{mk_{1}}z_{mk_{2}}\right]=\sum_{k=1}^{p}f_{ik}f_{jk}=\sigma_{ij}.$$

Especially when i=j, we have $E\left[x_{mi}^{2}\right]=\sigma_{ii}$. Then, we can obtain

(60) $$\begin{array}{cc} \; & E\left[n^{3}\mathrm{tr}\left(D_{S}^{3}\right)-3n\mathrm{tr}\left(GD_{S}\right)+2\mathrm{tr}\left(R\right)\right]=\sum_{m_{1}\neq m_{2}\neq m_{3}}^{n}\sum_{i=1}^{p}E\left[x_{m_{1}i}^{2}x_{m_{2}i}^{2}x_{m_{3}i}^{2}\right]\\ \; & =\sum_{m_{1}\neq m_{2}\neq m_{3}}^{n}\sum_{i=1}^{p}E\left[x_{m_{1}i}^{2}\right]E\left[x_{m_{2}i}^{2}\right]E\left[x_{m_{3}i}^{2}\right]=\sum_{m_{1}\neq m_{2}\neq m_{3}}^{n}\sum_{i=1}^{p}\sigma_{ii}^{3}\\ \; & =n\left(n-1\right)\left(n-2\right)\sum_{i=1}^{p}\sigma_{ii}^{3}=\tau_{c}^{-1}\mathrm{tr}\left(D_{\Sigma }^{3}\right). \end{array}$$

Furthermore, we have

(61) $$\begin{array}{cc} \; & E[n^{3}\mathrm{tr}\left(D_{S}S^{2}\right)-2n\mathrm{tr}\left(KS\right)-n\mathrm{tr}\left(GD_{S}E\right)+2\mathrm{tr}\left(RE\right)]\\ \; & =\sum_{m_{1}\neq m_{2}\neq m_{3}}^{n}\sum_{i,j=1}^{p}E\left[x_{m_{1}i}^{2}x_{m_{2}i}x_{m_{2}j}x_{m_{3}i}x_{m_{3}j}\right]=\sum_{m_{1}\neq m_{2}\neq m_{3}}^{n}\sum_{i,j=1}^{p}E\left[x_{m_{1}i}^{2}\right]E\left[x_{m_{2}i}x_{m_{2}j}\right]E\left[x_{m_{3}i}x_{m_{3}j}\right]\\ \; & =\sum_{m_{1}\neq m_{2}\neq m_{3}}^{n}\sum_{i,j=1}^{p}\sigma_{ii}\sigma_{ij}^{2}=n\left(n-1\right)\left(n-2\right)\sum_{i,j=1}^{p}\sigma_{ii}\sigma_{ij}^{2}=\tau_{c}^{-1}\mathrm{tr}\left(D_{\Sigma }\Sigma^{2}\right). \end{array}$$

□

Then, the unknown scalars $c_{1}$ and $c_{2}$ can be unbiasedly estimated by

(62) $$\begin{array}{cc} \gamma_{1} & =\tau_{c}\left[n^{3}\mathrm{tr}\left(D_{S}^{3}\right)-3n\mathrm{tr}\left(GD_{S}\right)+2\mathrm{tr}\left(R\right)\right], \end{array}$$

(63) $$\begin{array}{cc} \gamma_{2} & =\tau_{c}\left[n^{3}\mathrm{tr}\left(D_{S}S^{2}\right)-2n\mathrm{tr}\left(KS\right)-n\mathrm{tr}\left(GD_{S}E\right)+2\mathrm{tr}\left(RE\right)\right]. \end{array}$$

By plugging the estimates of quantities in (27) into (28) and (29), the unbiased estimates of $v(T_{1})$ and $v(T_{2})$ are given by

(64) $$\begin{array}{cc} \hat{v}\left(T_{1}\right)\; & =\left[\begin{array}{c} \frac{1}{p}\alpha_{2}-\alpha_{1}\\ \frac{np+p-4}{np}\beta_{1}+\frac{1}{p^{2}}\beta_{2}+\frac{p^{2}-2np+2}{np^{2}}\beta_{3} \end{array}\right], \end{array}$$

(65) $$\begin{array}{cc} \hat{v}\left(T_{2}\right)\; & =\left[\begin{array}{c} \alpha_{3}-\alpha_{1}\\ \frac{n+1}{n}\beta_{1}+\frac{1}{n}\beta_{3}+\frac{n+2}{n}\gamma_{1}-\frac{2n+4}{n}\gamma_{2} \end{array}\right]. \end{array}$$

By the Equations (16), (64), and (65), the unbiased estimates of the vectors $b(T_{i}),i=1,2$ are given by

(66) $$\hat{b}\left(T_{i}\right)=\hat{u}\left(T_{i}\right)+\hat{v}\left(T_{i}\right),i=1,2.$$

In addition, the constant *c* in (10) can be further calculated under Gaussian distribution, which is

(67) $$c=\frac{1}{n}a_{1}+\frac{1}{n}a_{2}.$$

Therefore, we can obtain that the unbiased estimate of *c* is given by

(68) $$\hat{c}=\frac{1}{n}\alpha_{1}+\frac{1}{n}\alpha_{2}=\tau_{a}\left(\frac{n-2}{n}\mathrm{tr}\left(S^{2}\right)+{\mathrm{tr}}^{2}\left(S\right)\right).$$

To sum up, by Equations (13), (66) and (68), we can obtain that the unbiased estimates of the loss functions $M_{T_{i}}\left(w|\Sigma \right)$ are given by

(69) $${\hat{M}}_{T_{i}}\left(w\right)=\frac{1}{2}w^{T}\hat{H}\left(T_{i}\right)w-2w^{T}\hat{b}\left(T_{i}\right)+\hat{c}.$$

### 3.3. Optimal Second-Order Stein-Type Estimators

For the target matrices $T_{i},i=1,2$, through replacing the objective function in (10) with its unbiased estimate given by (69), we further formulate the second-order Stein-type estimators as the following optimization problems:

(70) $$\begin{array}{cc} \min & \;\frac{1}{2}w^{T}\hat{H}\left(T_{i}\right)w-2w^{T}\hat{b}\left(T_{i}\right)+\hat{c}\\ s.t. & \;0\leq e_{1}w\leq 1,\\ \; & \;e_{2}w\geq 0. \end{array}$$

For i=1,2, the Hessian matrix of the objective function in (70) is $\hat{H}\left(T_{i}\right)$. By the following inequality

(71) $${\left(\mathrm{tr}{\left(S-T_{i}\right)}^{3}\right)}^{2}\leq \mathrm{tr}{\left(S-T_{i}\right)}^{2}\mathrm{tr}{\left(S-T_{i}\right)}^{4},$$

we can obtain that $\hat{H}\left(T_{i}\right)$ is positive definite. Therefore, the optimization problem (70) is a convex quadratic program. Furthermore, we can obtain the globally optimal solution by an efficient algorithm.

For the target matrices $T_{i},i=1,2$, by denoting the corresponding optimal tuning parameters as $w^{i}={\left(w_{1}^{i},w_{2}^{i}\right)}^{T}$, the optimal second-order Stein-type estimators can be expressed as

(72) $${\hat{\Sigma }}_{i}=S+w_{1}^{i}\left(T-S\right)+w_{2}^{i}{\left(T-S\right)}^{2}.$$

**Remark** **3.** *The proposed second-order Stein-type estimators are both well conditioned. Moreover, by taking the spectral decomposition, the SCM can be expressed as*

(73) $$S=U^{T}\Delta U,$$

*where $\Delta =\mathrm{diag}(\delta_{1},\ldots ,\delta_{p})$ is a diagonal matrix consisting of the eigenvalues and U is the corresponding unitary matrix. Then, the second-order Stein-type estimator ${\hat{\Sigma }}_{1}$ has the following spectral decomposition:*

(74) $${\hat{\Sigma }}_{1}=U^{T}\tilde{\Delta }U,$$

*where $\tilde{\Delta }=\mathrm{diag}\left({\tilde{\delta }}_{1},\ldots ,{\tilde{\delta }}_{p}\right)$ with*

(75) $${\tilde{\delta }}_{i}=\delta_{i}+w_{1}^{1}\left(\theta -\delta_{i}\right)+w_{2}^{1}{\left(\theta -\delta_{i}\right)}^{2}.$$

*where $\theta =\frac{\mathrm{tr}(S)}{p}$ is the mean of sample eigenvalues. Therefore, the proposed estimator ${\hat{\Sigma }}_{1}$ shrinks the sample eigenvalues by a nonlinear transformation whileretaining the sample eigenvectors.*

## 4. Numerical Simulations and Real Data Analysis

This section presents numerical simulations and two application examples to discover the performance of the proposed second-order Stein-type estimators. The proposed covariance matrix estimators for the target matrices $T_{1}$ and $T_{2}$ are denoted as QS-T1 and QS-T2, respectively. The control estimators include the Stein-type estimator LS-T1 for $T_{1}$ in [11], and the Stein-type estimator LS-T2 for $T_{2}$ in [12] and the nonlinear shrinkage estimator NS developed in [18].

### 4.1. MSE Performance

We assume that the actual distribution is N(0,Σ), where the following models generate the covariance matrix:

(1) Model 1: $\Sigma ={\left(\sigma_{ij}\right)}_{p\times p}$ with $\sigma_{ii}=1$ and $\sigma_{ij}=0.1^{\left|i-j\right|}$ for i≠j,
(2) Model 2: $\Sigma ={\left(\sigma_{ij}\right)}_{p\times p}$ with $\sigma_{ii}=\max\left\{50-i,0\right\}+2$ and $\sigma_{ij}=0.6$ for i≠j.

Under Model 1, the diagonal elements are equal to 1, and the off-diagonal elements are tiny. Therefore, the covariance matrix is close to a spherical matrix. Under Model 2, the diagonal elements are dispersive, and the off-diagonal elements correspond to weak correlations. Therefore, the covariance matrix is close to a diagonal matrix. We carry out random sampling in each Monte Carlo run and compute the Frobenius loss of each covariance matrix estimator. The MSE performance of each covariance matrix estimator is evaluated by averaging the Frobenius losses of $5\times 10^{3}$ runs.

**Figure 1:**
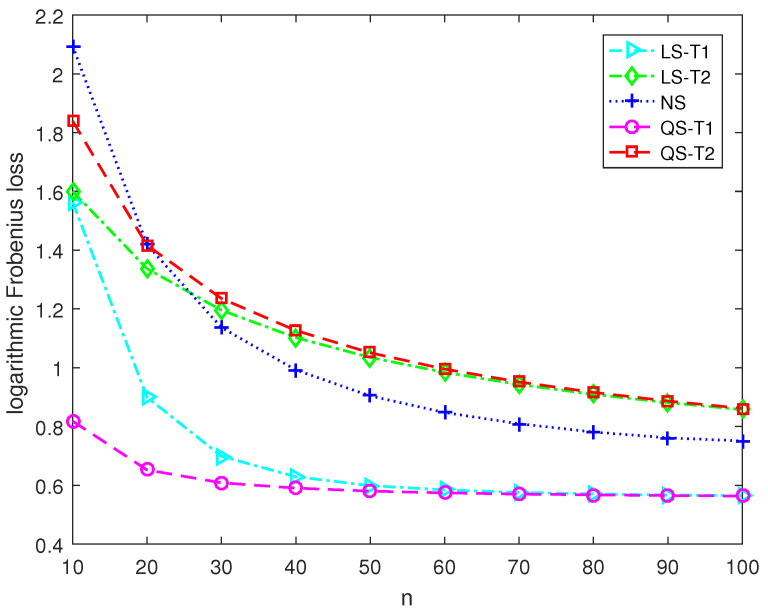
The logarithmic Frobenius losses of shrinkage estimators under Model 1 with p=180.

**Figure 2:**
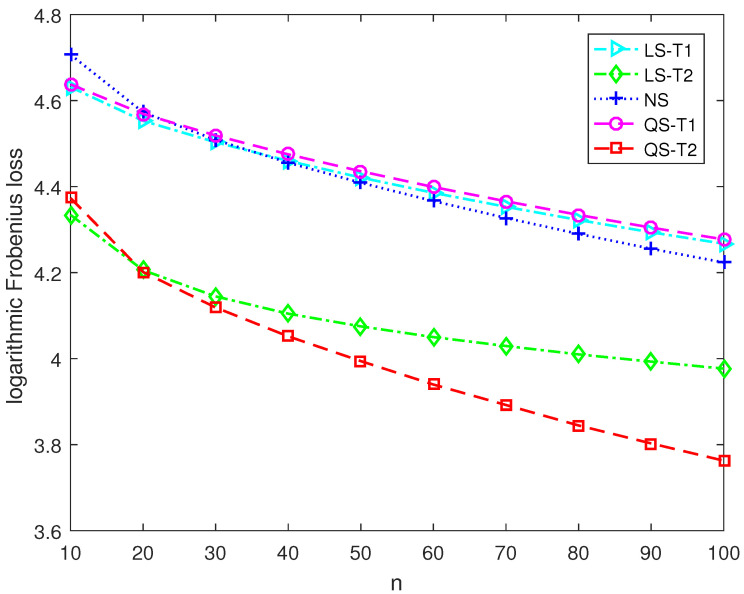
The logarithmic Frobenius losses of shrinkage estimators under Model 2 with p=180.

Figure 1 and Figure 2 report the logarithmic Frobenius loss of each estimator in large-dimensional scenarios where the dimension is 180 and the sample size varies from 10 to 100. Under Model 1, the Stein-type estimator LS-T1 and the second-order Stein-type estimator QS-T1 outperform the nonlinear shrinkage estimator NS and the shrinkage estimators LS-T2 and QS-T2, which employ the diagonal target matrix. Furthermore, the proposed second-order Stein-type estimator QS-T1 shows a significant advantage over the Stein-type estimator LS-T1, especially when the sample size is tiny. Similarly, under Model 2, the Stein-type estimator LS-T2 and the second-order Stein-type estimator QS-T2 perform better than the other three estimators. Moreover, the proposed estimator QS-T2 outperforms the corresponding Stein-type estimator LS-T2. Therefore, when the correct target matrix is employed, the second-order Stein-type estimators enjoy lower Frobenius losses than the linear and nonlinear shrinkage estimators in large-dimensional scenarios.

**Figure 3:**
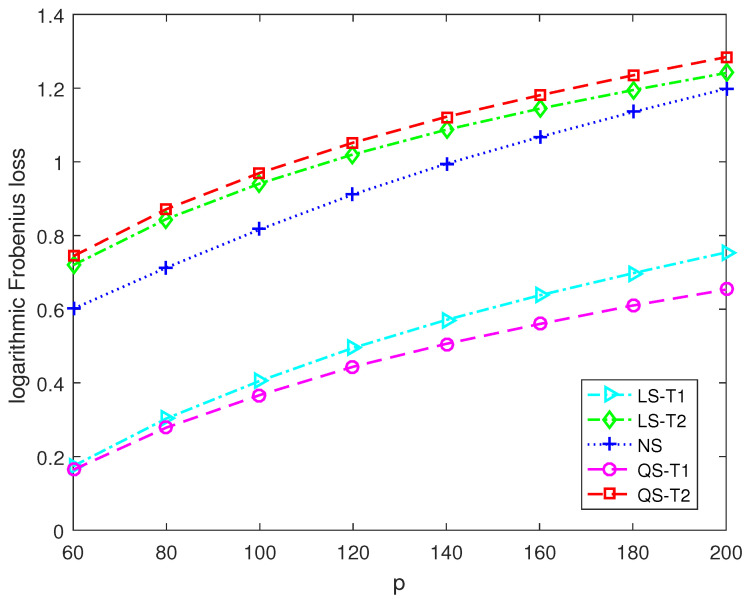
The logarithmic Frobenius losses of shrinkage estimators under Model 1 with n=30.

**Figure 4:**
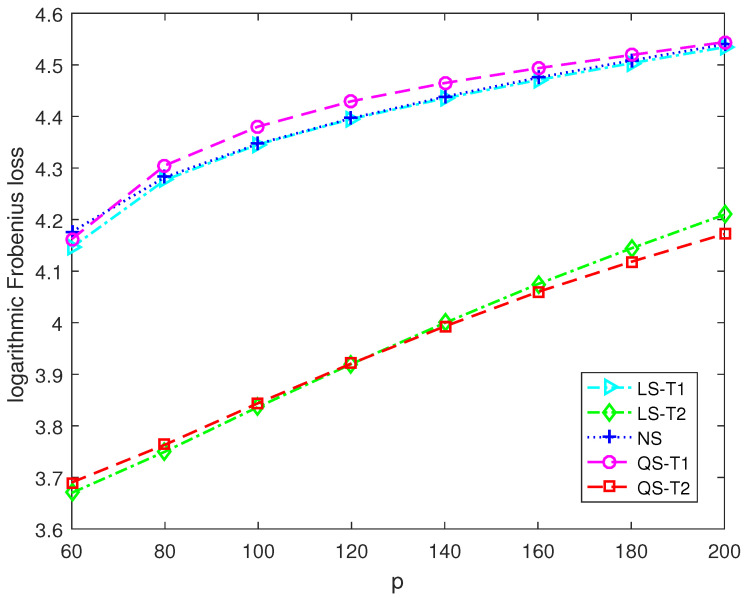
The logarithmic Frobenius losses of shrinkage estimators under Model 2 with n=30.

Figure 3 and Figure 4 report the logarithmic Frobenius loss of each estimator versus the dimension. The sample size is 30. We can find that the logarithmic Frobenius losses become more prominent as the dimension increases. In Figure 3, the actual covariance matrix is close to being spherical. The proposed second-order Stein-type estimator QS-T1 significantly outperforms the other estimators. In Figure 4, the actual covariance matrix is close to being diagonal. The proposed second-order Stein-type estimator QS-T2 enjoys lower Frobenius loss when the dimension exceeds 120.

The proposed second-order Stein-type estimators take significant advantage of the MSE performance over the linear and nonlinear shrinkage estimators, especially when the dimension is large compared to the sample size.

### 4.2. Portfolio Selection

In finance, assets with higher expected returns generally involve higher risks. Therefore, investors must constantly balance the expected return and the risk tolerance. The portfolio strategy is a popular way to reduce risk and enhance return. Therefore, portfolio selection plays a vital role in asset investment.

In 1952, Markowitz introduced the famous mean-variance optimization to determine the optimal portfolio weights [31]. Let m and Σ be the expectation and covariance matrix of the daily returns. For portfolio weight k, the variance of the portfolio is defined as $\sigma^{2}=k^{T}\Sigma k$ in the Markowitz framework. As short selling is forbidden, the Markowitz portfolio optimization is formulated as the following mean-variance problem:

(76) $$\begin{array}{cc} \min & \;k^{T}\Sigma k\\ s.t. & \;k^{T}m=r,\;k^{T}1=1,\;k\geq 0, \end{array}$$

where *r* is a given expected return. By, respectively, replacing m and Σ with their estimates $\hat{m}$ and $\hat{\Sigma }$, the optimal weight $k_{r}$ can be solved by efficient quadratic programming algorithm. It is obvious that the Markowitz optimization only depends on the estimates of the first and second moments of the daily returns. The sample mean and the SCM in the classic portfolio perform well in the portfolio risk measurement; however, the SCM becomes unstable as the number of stocks is large, resulting in significant property loss [32]. Therefore, a well-performed covariance matrix estimator is important in current portfolio selection [2,33].

In practice, we consider a portfolio consisting of p=95 highly capitalized stocks from the New York stock exchange with ticker symbols AA, ABT, AIG, AIR, ALL, AMD, AP, APA, AXP, BA, BAC, BAX, BEN, BK, BMY, C, CAT, CCL, CHK, CL, COP, CPE, CVS, CVX, D, DB, DD, DE, DNR, DVN, EAT, EME, EMR, EXC, FCX, FDX, FNMA, GD, GE, GILD, GLW, HAL, HD, HIG, HON, HPQ, IBM, INTC, ITW, JNJ, JPM, KMB, KO, L, LLY, LMT, LOW, M, MCD, MDT, MMM, MO, MRK, MRO, MS, NBR, NC, NE, NEE, NL, NNN, ODC, OXM, OXY, PCG, PEP, PFE, PG, RIG, SLB, SO, T, TGT, TRV, TXN, UNH, UNP, USB, VLO, VZ, WFC, WMT, WWW, X, XOM. The dataset X contains n=536 daily close prices from 11 November 2016 to 31 December 2018 collected via Yahoo! Finance at https://au.finance.yahoo.com/lookup?s=DATA, accessed on 10 November 2022. For each stock *i*, the daily close price is preprocessed as the daily return by

(77) $$\tilde{X}\left(i,j\right)=\frac{X(i,j+1)}{X(i,j)}-1,j=1,\ldots ,n-1.$$

The covariance matrix estimators LS-T1, LS-T2, NS, QS-T1, and QS-T2 are generated from the daily return $\tilde{X}$. For an expected return *r*, the realized risk is defined as $\sigma_{r}=\sqrt{k_{r}^{T}\hat{\Sigma }k_{r}}$. Next, we employ the realized risks, one key index to evaluate the portfolio, to verify the performance of the covariance matrix estimators.

**Figure 5:**
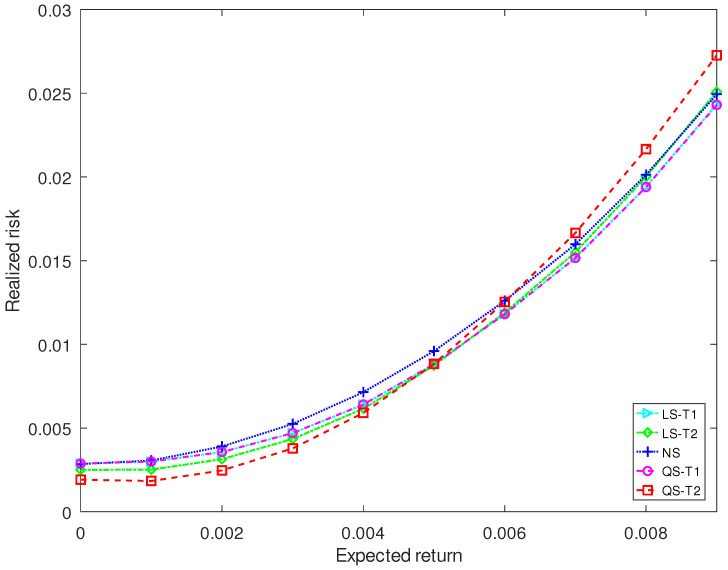
Realized risk of each covariance matrix estimator when the investment horizon contains 44 trading days.

**Figure 6:**
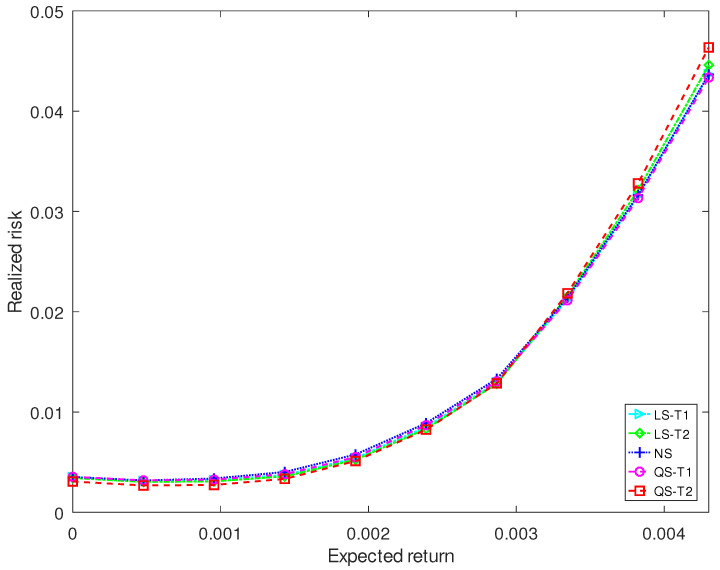
Realized risk of each covariance matrix estimator when the investment horizon contains 280 trading days.

Figure 5 and Figure 6 plot the realized risks of three kinds of shrinkage estimators for different investment horizons. For a short-term investment of 44 trading days (2 months), the proposed QS-T2 has the lowest realized risk when the expected return is less than 0.5% and has the highest realized risk when the expected return exceeds 0.6%. The proposed QS-T1 and the Stein-type estimator LS-T1 have the lowest realized risk when the expected return exceeds 0.5%. For a long-term investment of 280 trading days (13 months), the expected return of the five estimators becomes similar for the short-term investment.

**Figure 7:**
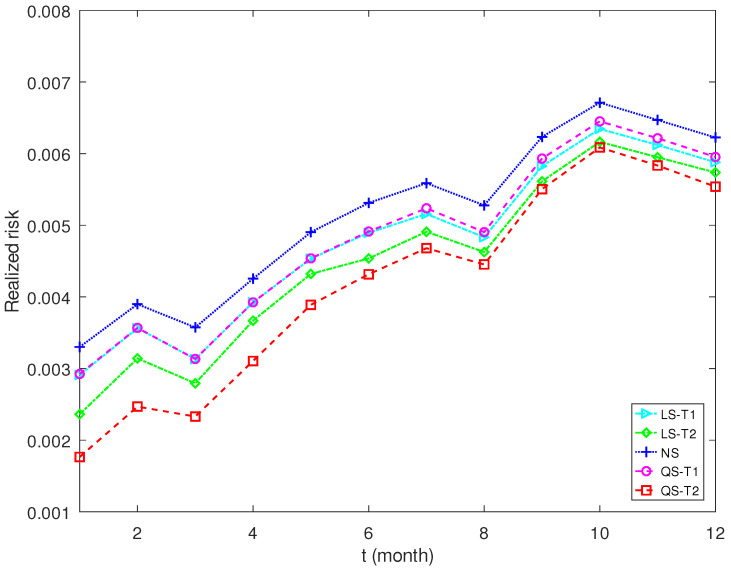
Realized risk of each covariance matrix estimator when the expected return is 0.2%.

**Figure 8:**
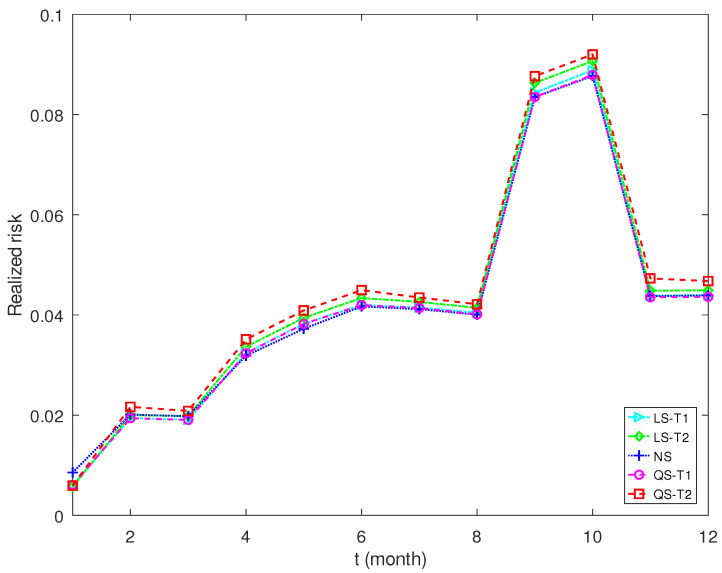
Realized risk of each covariance matrix estimator when the expected return is 0.8%.

Figure 7 and Figure 8 plot the realized risks of shrinkage estimators for different expected return levels. The proposed estimator QS-T2 enjoys the lowest realized risk for a low expected return. The nonlinear shrinkage estimator NS has the highest realized risk. However, the proposed estimator QS-T2 performs worst when the expected return becomes high. The proposed estimator QS-T1, together with NS and LS-T1, performs best in this scenario.

The proposed second-order Stein-type estimator QS-T2 enjoys good portfolio selection for short-term investment and prudent return cases. The proposed second-order Stein-type estimator QS-T1 is recommended in long-term investment and high-return scenarios.

### 4.3. Discriminant Analysis

We further discover the performance of the second-order regularized estimators in small-sample-size situations. The Parkinson’s data are collected on the website https://archive-beta.ics.uci.edu/, accessed on 10 November 2022. p=160 biomedical voice attitudes are measured from $n_{1}$ patients and $n_{2}$ healthy individuals. Let $\hat{\Sigma }$ be the pooling estimator based on a certain estimation strategy. We use the following quadratic discriminant rule $M={\left(x_{i}-\bar{x}\right)}^{T}{\hat{\Sigma }}^{-1}\left(x_{i}-\bar{x}\right)$ to make a diagnosis for each $x_{i}$. *M* denotes the Mahalanobis distance between individual $x_{i}$ and the sample center $\bar{x}$. The individual $x_{i}$ is classified as Parkinson’s patient if $x_{i}$ is closer to the sample center of patients in the sense of Mahalanobis distance.

Table 1 reports the classification accuracy rates of different estimators. We can see that the Stein-type estimators LS-T1, LS-T2, QS-T1, and LS-T2 perform better than NS. Moreover, the accuracy rate of the Stein-type estimators becomes larger as the sample size increases. Moreover, the proposed second-order Stein-type estimator QS-T2 enjoys the largest accuracy rate when n≤40, and the Stein-type estimator LS-T2 has the best performance when n≥45. Therefore, we can see that the proposed second-order Stein-type estimation performs better than the classic Stein-type estimation in this application.

## 5. Conclusions and Discussion

This paper investigated the problem of estimating a large-dimensional covariance matrix. Motivated by Stein’s strategy, we developed a novel strategy named the second-order Stein-type estimation. The proposed estimator is expected to be positive definite in the form of a quadratic binomial of the SCM and the target matrix. Firstly, we specified the spherical and diagonal targets in the second-order Stein-type regularization. The mean squared errors were, respectively, obtained for the two targets. Secondly, we unbiasedly estimated the two mean squared errors under the Gaussian distribution. Thirdly, the optimal parameters were obtained by solving the convex quadratic programming. The optimal second-order Stein-type estimators were obtained for the two target matrices. Finally, we verified the performance of the proposed estimators in numerical simulations and real data applications.

It is worth mentioning that the second-order Stein-type estimators were proposed under Gaussian distribution. In practical applications, the data may often deviate from the Gaussian distribution. Therefore, the problem of investigating the second-order Stein-type regularization under non-Gaussian distributions remains open and important.

## Figures and Tables

**Table 1:** The classification accuracy rate of Parkinson’s data.

$n_{2}$	15	20	25	30	35	40	45	50
LS-T1	0.5165	0.5530	0.5646	0.5826	0.5870	0.5956	0.5981	0.6054
LS-T2	0.6262	0.6459	0.6607	0.6714	0.6796	0.6892	**0.6918**	**0.6971**
NS	0.4931	0.4842	0.4701	0.4688	0.4544	0.4458	0.4285	0.4304
QS-T1	0.5124	0.5465	0.5600	0.5761	0.5833	0.5938	0.5953	0.6025
QS-T2	**0.6336**	**0.6539**	**0.6678**	**0.6747**	**0.6840**	**0.6902**	0.6916	0.6957

## Data Availability

All data included in this study are available upon request by contact with the first author.
